# Exploring the model of PC12 apoptosis induced by OGSD/R through *in vitro* experiments

**DOI:** 10.18632/oncotarget.21623

**Published:** 2017-10-04

**Authors:** Yanqing Sun, Wei Zhu, Shengyuan Zhou, Zhiwei Wang, Xiongsheng Chen, Lianshun Jia

**Affiliations:** ^1^ Department of Spine, Changzheng Hospital, Second Military Medical University, Shanghai 200003, China

**Keywords:** oxygen-glucose-serum deprivation/restoration, PC12, apoptosis, ischemia reperfusion

## Abstract

**Aims:**

To explicit cell apoptosis trend in PC12 oxygen-glucose-serum deprivation/restoration (OGSD/R) model and provide experimental bases for neural cell simulation in ischemia reperfusion injury *in vitro*.

**Methods:**

OGSD/R model was constructed using the passage PC12 cells *in vitro*. The profile of cell apoptosis was estimated by DAPI staining, Annexin V-FITC and terminal deoxynucleotidyl transferase dUTP nick-end labeling (TUNEL) assay, as well as the levels of apoptosis-related proteins, including procaspase-3 and caspase-12.

**Results:**

PC12 apoptosis was induced by OGSD and aggravated after restoration. CCK8 assay indicated that cell activity reached minimum after 1h of oxygen-glucose-serum restoration (OGR). DAPI staining suggested that apoptosis was the most serious after 1h of OGR, causing apoptotic cell nucleus pyknosis, particle spot formation, and fracture of cells with serious apoptosis forming pieces, and nucleus disintegration. The percentage of apoptotic cells exhibited increased trend after restoration, and reached the highest at 1h of OGR. Moreover, the expression of procaspase-3 and caspase-12 were extremely enhanced after OGD, especially 1h after OGR.

**Conclusions:**

PC12 apoptosis is induced by OGSD and aggravated after restoration. The apoptosis of PC12 reaches the highest at 1h after OGR, which may provide experimental bases for spinal cord ischemia reperfusion injury treatment.

## INTRODUCTION

Spinal cord injury frequently causes paralysis of the limbs, even causes severe death. The annually increasing number of patients with spinal cord injury has become a major economic burden of the world due to lack of effective treatments by far. According to relevant reports by the United States, over 7 billion dollars are spent on the treatment and care of patients with spinal cord injury averaging 31.7 years of age with a peak age of onset at 15-25, who require long-term treatment and care [1]. Thus it is of great significance to explore the etiology of spinal cord injury.

Reperfusion is the main treatment for patients with spinal cord injury. However, growing evidences have demonstrated that reperfusion can induce the production of free radical, toxin, and inflammatory, thus aggravating cell apoptosis. For example, NO represents an important free radical, which has been reported to be involved in various cell apoptosis through different ways thus leading to diverse diseases. It is generally accepted that the severe apoptosis of mucosal epithelial cells induced by over accumulation of NO and subsequent local cytotoxicity is an important pathogenic mechanism of necrotizing enterocolitis [2]. Therefore, to improve the treatment of spinal cord injury is urgently needed. The pathological processes of spinal cord injury include primary and secondary injury. The necrotic nerve cells resulting from primary injury can not be repaired, and secondary injury occurs based on primary injury. Neuronal apoptosis and necrosis caused by extracellular toxin, free radical, and inflammatory mediator, which are produced by ischemia reperfusion injury and inflammatory factors, are pathological bases of secondary spinal cord injury [3]. Consequently, the prevention of neuronal apoptosis plays a crucial role in the protection and functional recovery of nerve cells after spinal cord injury.

PC12, a set of cell lines isolated from adrenal medulla pheochromocytoma of rat, is generally characterized by neuroendocrine cell and passage; thus, the differentiated PC12 can be used as *in-vitro* cell model to investigate the physiological and biochemical functions of neuronal cells [4]. It has been reported that oxygen-glucose-serum deprivation/restoration (OGSD/R) can induce the apoptosis of spinal cord astrocytes through activating the endoplasmic reticulum to stress relevant apoptosis pathway [5]. In this process, the related apoptosis protein in endoplasmic reticulum (caspase-12) is activated, further causing apoptosis via a series of cascade reactions [5]. Procaspase-3 represents a common apoptosis-related protein, OGSD/R can also induce up-regulation of caspase in PC12 cells [6]. It had been reported that OGD/R could induce PC12 apoptosis varies from 2-24h [7–11]. There was no conclusion on this issue.

In the current study, we simulated the pathophysiological process of spinal cord ischemia reperfusion injury in PC12 cells to establish a stable injury model of OGSD/R. The related morphological observation and apoptosis indexes detection were performed to ascertain the time window of aggravating injury after OGSD/R in PC12 cells. The present study aimed to further provide experimental bases for drug intervention of apoptosis.

## RESULTS

### Morphological observation of apoptosis and apoptotic count (DAPI staining)

**Figure 1:**
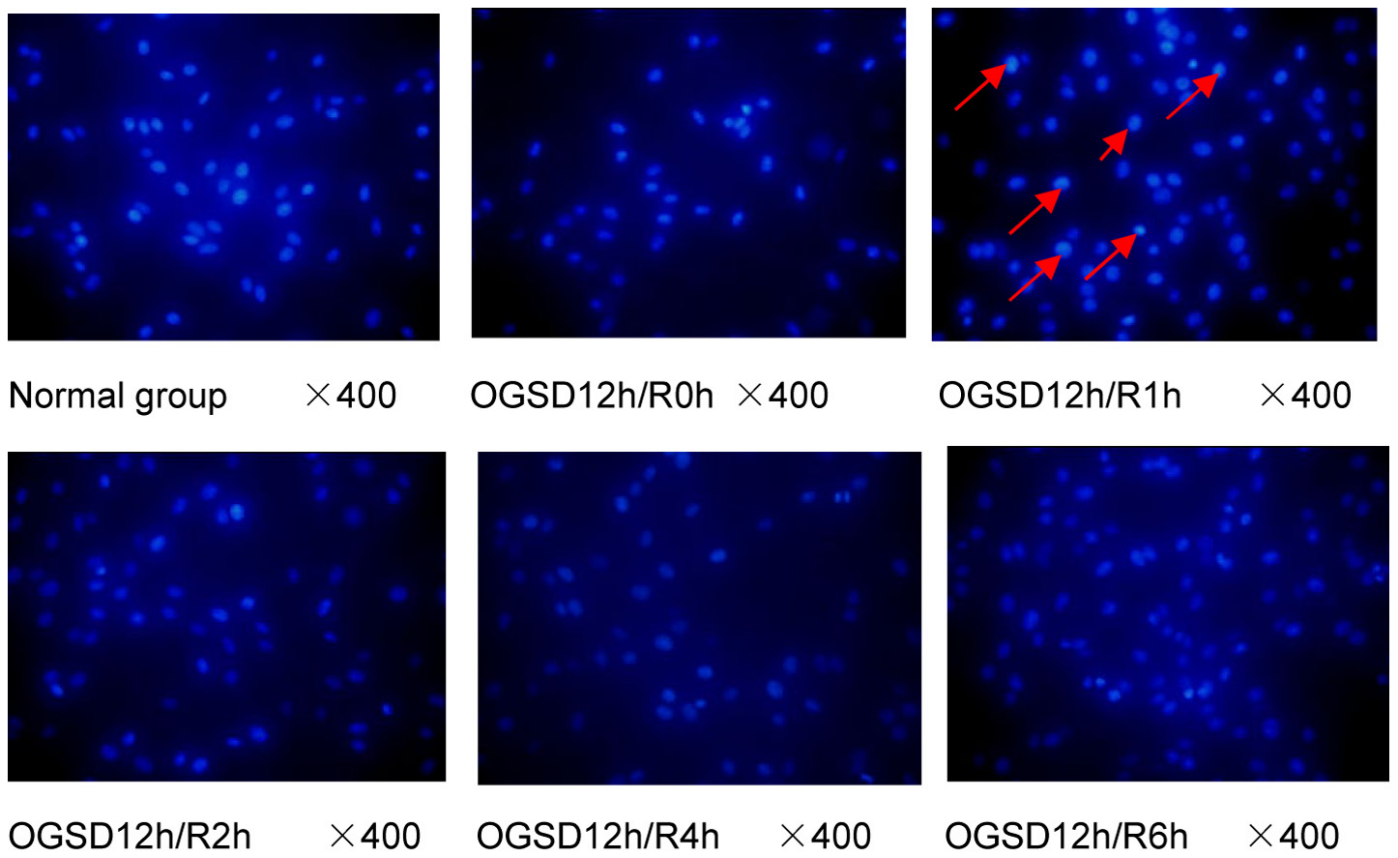
The apoptosis of a larger number of PC12, karyopyknosis, and partial nuclear fragmentation are found in group OGSD12h/R1h

According to the cell morphological characteristics and light intensity under microscope, the results were grouped into 3 grades. Grade I: cell nuclei presents wavy or crease, and only mild karyopyknosis was observed under microscope. The figures presented weak light, representing mild cell apoptosis; Grade IIa: The nuclear staining presents deep and patchy, and obvious karyopyknosis as well as high light was observed under microscope, revealing moderate cell apoptosis; Grade IIb: apoptotic bodies were observed, and the light was glaring under microscope, suggesting severe cell apoptosis. DAPI staining of cells and cells in normal group was performed with OGSD 12h/R at 0h, 1h, 2h, 4h, and 6h. OGR caused aggravation of apoptosis and karyopyknosis, forming a number of particle spots. Severe apoptosis of the nucleus broke into pieces, which resulted in the disintegration of nucleus (Figure 1). The figure for OGSD 12h/R at 1h exhibited the highest light, obvious karyopyknosis, and lots of particle spots, representing the highest apoptotic rate.

### Results of cell activity detection (CCK8)

**Figure 2:**
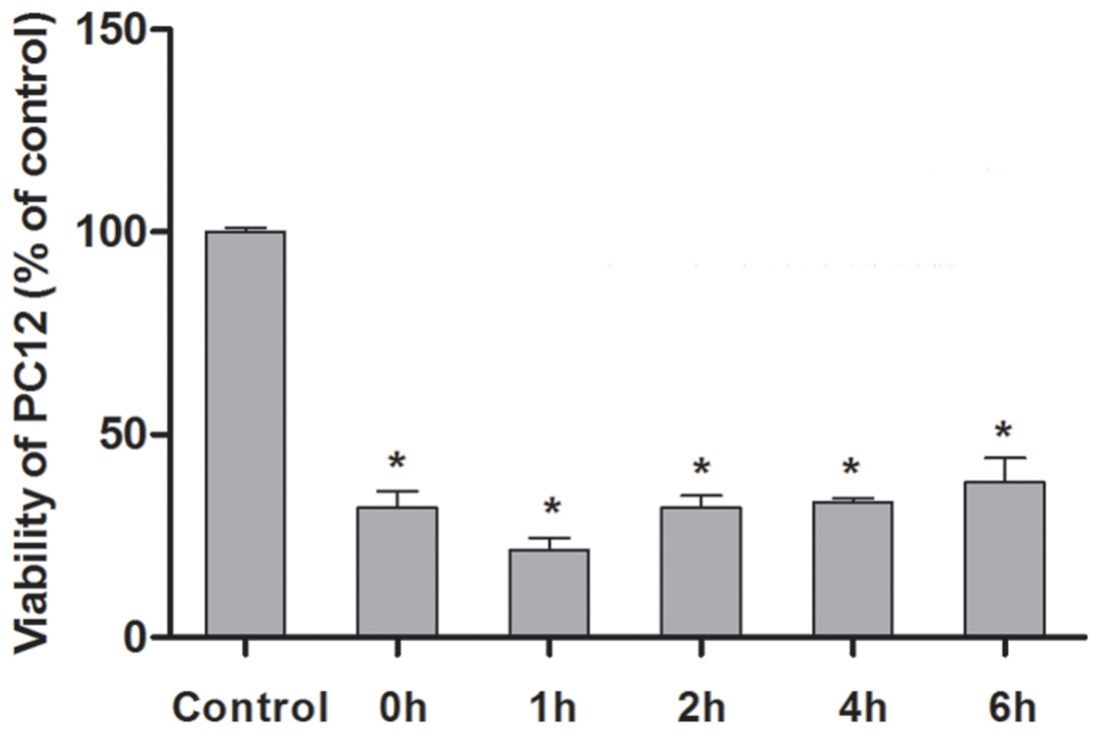
Cell activity variation at different time points after OGSD 12h/R Compared with control group, the cell activity obviously decreased after OGSR (*P*<0.05), and the cell activity exhibited the lowest after 1h of OGSR. ^*^: suggested *P*<0.05.

CCK8, which is convenient to use with high-speed detection, higher sensitivity, better repeatability than MTT, and less toxic to cells, can better reflect the number of active cells. Its light absorption value can be measured at 450 nm by ELIASA. The current experiment utilized CCK8 detection kit to test cell activity of group OGSD 12h/R 0h, 1h, 2h, 4h, and 6h, finding that cell apoptosis reached the highest at OGSD 12h/1h, which was statistically significant compared with other groups (*P*<0.05) (Figure 2).

### Flow cytometry examination

**Figure 3:**
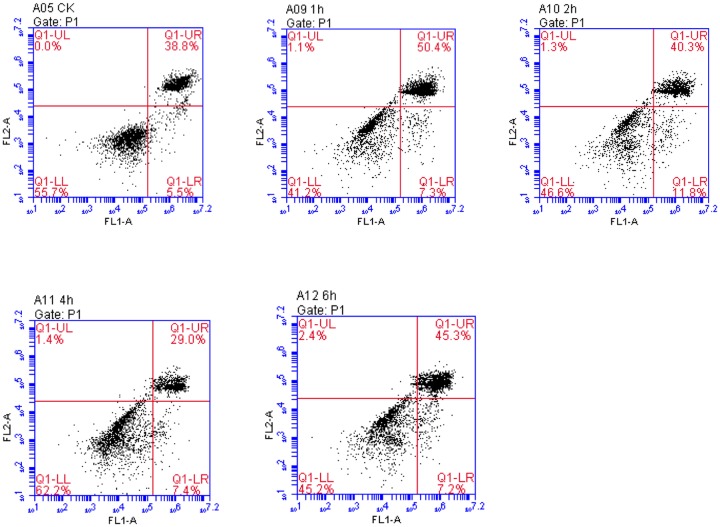
Comparison of early, late, and total apoptosis at different time points after OGSR Q1-UL represented the died cells; Q1-LL represented the percentage of normal cells; Q1-UR exhibited the percentages of late apoptotic cells; Q1-LR represented the percentage of early apoptotic cellls. The highest percentage of apoptotic cells was found at the OGSR 12h/1h than that at other time points (*P*<0.05).

The flow cytometry method, which could make rapid, accurate, and quantitative analysis of apoptosis, was applied to examine the situation of apoptosis and to calculate the percentage of apoptosis at different time points. The results showed that the total apoptosis rate at 1h of OGSR was apparently higher than that at other time points and the differences were statistically significant (*P*<0.05), which was consistent with the observed results of cell morphology and detected results of cell activity (Table 1 and Figure 3).

**Table 1:** Comparison of apoptosis rates at different time points after OGSD 12h/R (x¯ ±SD)

Apoptosis rate (%)	0h	1h	2h	4h	6h
Late apoptosis	38.0±5.1	50.8±4.0	40.5±3.2	29.5±4.4	45.6±6.7
Early apoptosis	10.9±2.3	8.3±2.6	14.4±1.0	10.2±2.6	9.9±4.3
Total apoptosis	48.9±6.3	59.1±5.2	54.8±3.9	39.7±4.0	55.6±3.2

The total apoptosis rate at 1h of OGSR is apparently higher than that at other time points, and the differences are statistically significant (*P*<0.05).

### TUNEL assay

**Figure 4:**
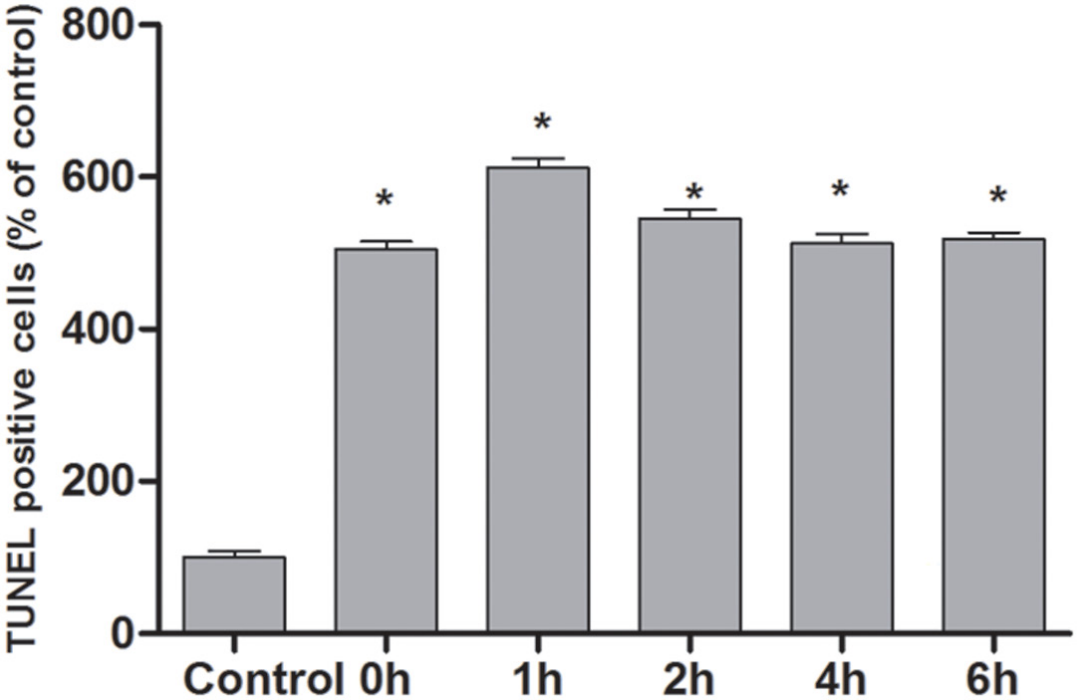
The profiles of cell apoptosis at different time points estimated by TNUEL assay The DNA fragmentation was increased in the groups received OGSR compared with the control group (*P*<0.05) and was higher at 1h of OGSR than that at other time points (*P*<0.05).

According to the TUNEL assay, the apoptosis of cells at different time points after OGSR was estimated. As shown in Figure 4, the increased DNA fragmentation was observed in the groups received OGSR compared with the control group (*P*<0.05). In addition, the TUNEL positive cells at 1h of OGSR was significantly higher than that at other time points (*P*<0.05). These results were in accordance with the results of flow cytometry examination and cell activity assay.

### Expression of caspase-12 apoptosis protein

**Figure 5:**
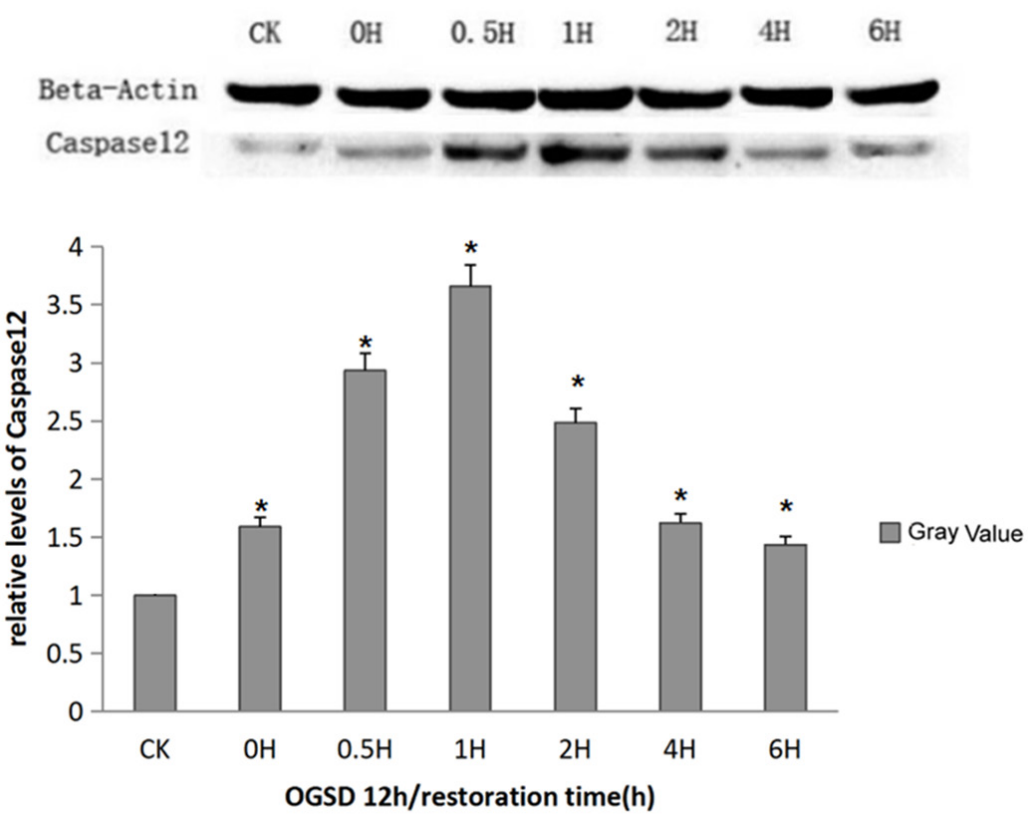
Caspase-12 protein expression at different time points Caspase-12 content was increased at each time point after OGSR compared with control group (*P*<0.01), and was obviously higher at the OGSD 12h/1h than that at other time points (*P*<0.01).

The endoplasmic reticulum stress could lead to destruction of intracellular homeostasis, activate the activity of caspase-12, further give rise to a series of cascade reactions, and eventually cause apoptosis. The expression of caspase-12, which was extremely low in normal cultured PC12, enhanced after the stimulation of OGSD/R and further enhanced after OGSR. The enhancement of caspase-12 at 6h of OGSD 12h/R was considered to be caused by natural apoptosis (Figure 5).

### Expression of procaspase-3 apoptosis protein

**Figure 6:**
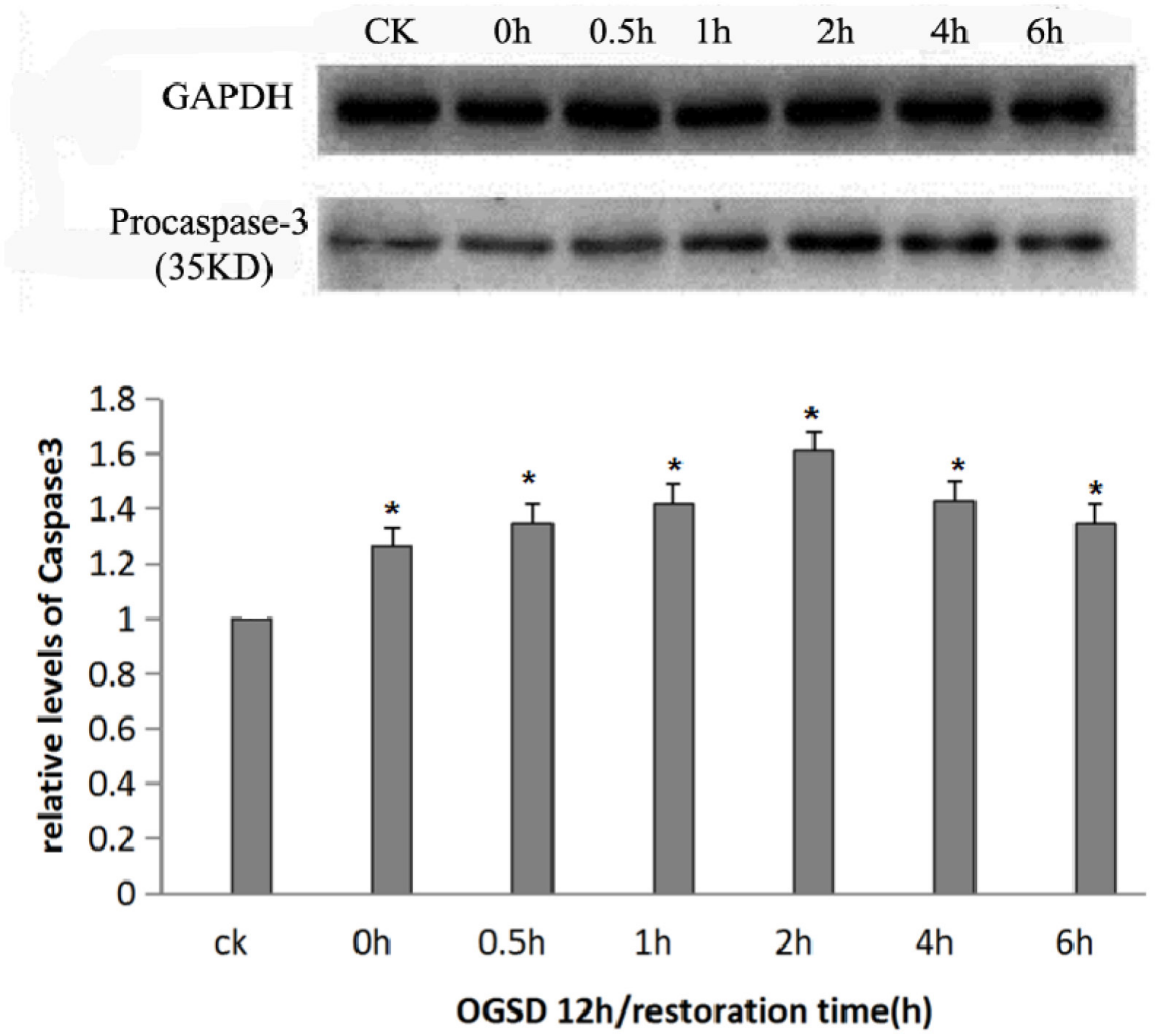
Procaspase-3 protein expression at different time points Procaspase-3 content was increased at each time point after OGSR compared with control group (*P*<0.05), and was significantly higher at OGSD 12h/2h than that in the other groups (*P*<0.05).

Caspase, which is a group of cysteine proteinase, plays an important role in different stages of apoptosis. It has no activity in normal cells, but can induce apoptosis after being activated. Caspase-3 plays a pivotal role in cell apoptosis. Procaspase-3, the zymogen form of caspase-3, is the effector of cell apoptosis signaling pathway. Procaspase-3 could induce degradation of DNA via activating endonuclease, thus leading to cell apoptosis [12]. This experiment found that procaspase-3 content, which was relatively low in normal cultured PC12, enhanced after being stimulated by OGSD/R and was apparently higher at 2h of OGSR compared with that at other time points (*P*<0.05), suggesting that OGSD/R could aggravate PC12 apoptosis induced by OGSD (Figure 6).

## DISCUSSION

So far, there are two commonly used ischemia and hypoxia models *in vitro*. The first one is physical hypoxia model, which cultures cells under the conditions of hypoxia with the absence of glucose and other nutrients. Physical hypoxia model is the most frequently used model at present, which applies tri-gas incubator to make cells to be at anoxic condition in the solution of low oxygen and high nitrogen, and adopts serum-free medium with low glucose in the meantime. The model requires high-level equipment. The experiment has also been carried out by the self-made hypoxia box by far, but it is difficult to control experimental conditions. The second one is chemical hypoxia model, which uses the toxic substances produced after ischemia such as glutamic acid and glycine to intervene in damage model, or uses reagents such as cobalt chloride, sodium hydrosulfite, and sodium persulfate to simulate anoxic environment. It is characterized by stable experimental conditions as well as simple and convenient operations. However, chemical hypoxia model is less common than physical hypoxia model. Cell ischemia reperfusion injury model can be jointly established by one or more of the above models. Our experiment was conducted to simulate ischemia reperfusion injury *in vitro* through OGSD/R, to keep cells at anoxic condition in the solution of low oxygen and high nitrogen, and to detect various indexes via applying glucose-free medium without serum after OGSR. Although there were several influencing factors including pH value of medium, ionic concentration, and hemorheology, this model exhibited stable results and good repeatability, moreover the operations were simple and convenient. The model used in this study could effectively simulate the pathological processes of progressive depletion of intracellular oxygen energy and after oxygen restoration [5].

Wu Xiaomei et al. simulated cell hypoxia model using the self-made sealing device used for hypoxia and DMEM medium without serum, finding that the activity of mouse cortical neurons gradually decreased after 4-12h of hypoxia, with gradually increased apoptotic cells and aggravated cellular damages as the extension of time [13]. In the pretreatment study of hypoxia of mouse hippocampal neuronal cells, Wu LY et al. informed that the damage was further aggravated after 4h of neuronal cell hypoxia and 24h of oxygen restoration [14]. In the present experiment, we found that OGSD could induce PC12 apoptosis and the cell activity further decreased after OGSR, with aggravated apoptotic injury and apoptotic peak appearing at 1h of OGSR. To further confirm the apoptosis of cells after OGSR, TUNEL assay was conducted and found that the percentage of apoptotic cells was increased in the groups received OGSR compared with the control group and reached the highest at 1h of OGSR. Several reasons might be responsible for the divergence, such as the procedures for OGSR model construction, the type of used cells, as well as the detection method. In addition, we also found that compared with control group, the percentage of apoptotic cells exhibited the lowest level 4h after reperfusion. That might be a promising time for spinal cord ischemia reperfusion. However, the related results were rarely reported in the previous studies. Further analyses were required to address the related issues.

The full name of caspase is aspartate proteolytic enzyme containing cysteine, which is the key apoptosis-related protease. Caspase-12, which exists on the endoplasmic reticulum membrane in the form of proenzyme (procaspase-12) before being activated, is a key factor to mediate the endoplasmic reticulum stress apoptosis [15]. When cells start apoptotic response, the accumulation of intracellular calcium can induce calpain to transfer from the cytoplasm to cell membrane, thus shearing procaspase-12 and activating caspase-12 as well as caspase-3 through a series of cascade reactions, further resulting in cell apoptosis [16, 17]. According to the functions and sequence homology of caspase family, it can be divided into three types, which are apoptotic promoter, apoptotic execution factor, and inflammatory mediator [18]. Caspase-3 is the protease for executing apoptosis, and a LETD175S sequence exists in the large and small subunits of caspase-3, which makes apoptotic promoter accurately trigger the execution factor target in apoptotic cascade reactions [19]. The promoters including caspase-8, -9, -10, -2 shears the execution factors including caspase-3, -6, -7, further degrading structural protein, signal molecule, and DNA repair enzyme [20]. After being activated, caspase-6, -7 can also further activate procaspase-3, and the activated procaspase-3 is able to successively shear procaspase-6, -9, and itself as well [21]. This positive feedback regulation eventually leads to apoptosis. Previous studies demonstrate that the abnormal proliferation of nerve cells and megalencephaly in the central nervous system are closely associated with caspase-3 in knockout mouse model, and these abnormal proliferation is primarily located in the central nervous system [22]. Recent studies have suggested that the control of caspase-3 activity can dramatically increase the proliferation and metastasis of neural precursor cells in the subventricular zone of mouse and can also improve the nerve function reconstruction after traumatic brain injury as well as the regeneration capacity of neuronal cells, all of which reveal that caspase-3 can promote endogenous neuronal apoptosis induced by trauma [23]. In the current study, western-blot method was employed to test the level of intracellular apoptosis protein, pointing that the levels of procaspase-3 and caspse-12 were extremely low in normal PC12 but increased after being stimulated by OGSD, and further enhanced after 1-2h of OGSR with basically consistent expression trends, which indicated that the apoptotic injury of PC12 induced by OGSD/R was time-independent and could better simulate the pathophysiological process of spinal cord ischemia reperfusion injury. In the process of OGSD/R inducing PC12 apoptosis, caspase-12 was activated and its expression was time-independent. Additionally, caspase-12 is a key factor that regulated endoplasmic reticulum to stress apoptosis pathway, informing that OGSD/R may induce PC12 apoptosis via activating endoplasmic reticulum stress related apoptosis pathway, but its specific mechanism needs further exploration.

In conclusion, low concentration of oxygen and sugar can induce cell apoptosis in spinal cord ischemia, and reperfusion may aggravate injury. The cell apoptosis researches the highest at 1h after reperfusion. The time for reperfusion is critical for spinal cord ischemia reperfusion injury treatment.

## MATERIALS AND METHODS

### Experimental cells culture and grouping

PC12 was purchased from Shanghai cell bank of Chinese Academy of Sciences. PC12 was revived and then cultivated with 10% FBS 1×P.S. 1640 culture medium (C11875500BT, GIBCO, USA) at 37°C as well as incubator with 5% CO_2_. 10 nM 7S nerve growth factor (Sigma-Aldrich, St. Louis, MO, USA) was added to the culture medium to induce PC12 cells to neuronal PC12 cells. The cells were co-cultured with the nerve growth factor for 3 days. When the cell density reached about 80%, the cells were cleaned with PBS (pH 7.4), and digested with 1ml 0.25% pancreatin, which was suctioned and abandoned after affecting for 2-3min, then 3ml preheating DEME complete medium at 37°C was added. The bottle bottom was gently blown until the cells were blown down. High-speed centrifuge was used to remove cell suspension. Cell precipitation was collected and added 6ml DEME complete medium for the resuspension of cells. The cell suspension was inoculated into 3 new culture bottles (2ml/bottle) and replenished with 3ml DEME complete medium, which was cultured with 5% CO_2_ at 37°C and saturated humidity. The subcultured cells were divided into control group (normal cultured PC12) and case group (OGSD 12h/R 0-6h).

### Establishing OGSD/R model *in vitro*

PC12 in logarithmic phase were inoculated in 24 pore plate (1×10^4^/pore) and cultured overnight in complete culture medium. Then the normal culture medium was removed, and the cells were cleaned twice with D-hank's (pH 7.4) and placed in sugar-free 1640 culture medium (11879-020, GIBCO, USA) without serum. The culture plate was placed in tri-gas incubator (95%N_2_+5%CO_2_, O_2_<1%, Forma Scientific) and was substituted for normal culture medium after taking out the cell after 12h, then it was observed at 37°C for 0-6h. During OGD, an oxygen electrode (East China University of Science and Technology, Shanghai, China) was applied to monitor the oxygen concentration of the medium. Cellular morphology was observed. CCK8 assay was used to estimate the cell proliferation, and cell apoptosis was evaluated by DAPI staining, Annexin V-FITC and terminal deoxynucleotidyl transferase dUTP nick-end labeling (TUNEL) assays. Additionally, the protein levels of procaspase-3 and caspase-12 were also detected at different time points of OGSR to investigate cell apoptosis.

### DAPI staining method

PC12 in logarithmic phase were inoculated in 24 pore plate (1×10^4^/pore) and cultured overnight in complete culture medium with cell attachment, which was replaced with sugar-free 1640 culture medium without serum the next day. Meanwhile, the gas ratio of tri-gas incubator was set to 5% CO_2_, 94% N_2_, and 1% O_2_, cultured without oxygen for 12h. The culture condition was changed after 12h. The cells were removed to 1640 culture medium with high glucose and were cultured with OGR for 0h, 1h, 2h, 4h, and 6h respectively. Cells in each group were collected to be rinsed twice with PBS. The membrane was ruptured with 0.1% Triton X-100 (Santa Cruz, USA) for 10min and washed twice with PBS, then was added 10 ng/ml DAPI solution (Sigma, USA) for lucifugal incubation at room temperature for 20min, and washed twice with PBS. The detection was performed with fluorescence microscope (BX43, OLYMPUS, Japan) under Uv channel for 30ms of exposure. Five fields of view were randomly taken to be photographed at 400 times.

### Detection of cell activity in each group by CCK8 at different time points

Cells in each group cultured with OGSR for 0h, 1h, 2h, 4h, and 6h respectively were collected and were added 10ul CCK8 (Dojindo, Kumamoto, Japan) detection solution to every pore. The culture plate was hatched in the incubator for 1h. ELIASA (CST, USA) was applied to measure the absorbancy at 450 nm. OD value was recorded and cell survival rate was calculated.

### Flow cytometry examination

Cells in each group cultured with OGSR for 0h, 1h, 2h, 4h, and 6h respectively were collected and rinsed twice with PBS, then were resuspended with 100ul PBS (pH 7.4). The percentage apoptotic cells was estimated by annexin V-FITC/PI double staining, which was carried out in accordance with the manufacturer's instructions of the Annexin V-FITC Apoptosis Detection Kit (BD, SanJose, CA, USA). The final rate of apoptosis was measured by BD FACSCantoII flow cytometer (BD Biosciences, USA) with BD FACSDiva (BD Biosciences, USA) software.

### Terminal deoxynucleotidyl transferase dUTP nick-end labeling (TUNEL) assay

To further confirm the apoptosis in PC12 cells in each group cultured with OGSR for 0h, 1h, 2h, 4h, and 6h, the TUNEL assay was performed in the current study with an *In Situ* Cell Death Detection Kit (Roche, Mannheim, Germany) following the manufacturer's protocols.

### Expression of procaspase-3, caspase-12 apoptosis protein level

Cells in each group cultured with OGSR for 0h, 0.5h, 1h, 2h, 4h, and 6h were collected via centrifugation at 2000rpm for 1min, and the medium was removed. The collected cells were washed using PBS buffer (pH 7.4) for three times. Then cell lysate was added to the cells, and the protein samples were isolated after centrifugation at 12000rpm for 10 min. Western-blot was adopted to detect the expression of procaspase-3, caspase-12 contents and to compare the expression of protein levels at different time points. The antibody against procaspase-3 (1:1000) and anti-caspase-12 (1:1000) were both purchased from Cell Signaling Technology (Beverly, MA, USA). In addition, the HRP-conjugated secondary antibodies (1:3000, ZSGB-BIO, Beijing, China) was used in this analysis. The relative expression level of the proteins were evaluated by the gray values of the bands.

### Statistical analysis

Each experiment was repeated in triplicates, and the data were expressed by x¯ ±SD, and data processing as well as statistical analysis were carried out with SPSS 13.0. Single factor analysis of variance method was introduced for statistical analysis, and *P*<0.05/*P*<0.01 represented obviously significant difference.

## References

[1] McDonald JW, Sadowsky C (2002). Spinal-cord injury. Lancet.

[2] Talavera MM, Nuthakki S, Cui H, Jin Y, Liu Y, Nelin LD (2017). Immunostimulated arginase II expression in intestinal epithelial cells reduces nitric oxide production and apoptosis. Front Cell Dev Biol.

[3] Lu J, Ashwell KW, Waite P (2000). Advances in secondary spinal cord injury: role of apoptosis. Spine.

[4] Shafer TJ, Atchison WD (1991). Transmitter, ion channel and receptor properties of pheochromocytoma (PC12) cells: a model for neurotoxicological studies. Neurotoxicology.

[5] Zhang A, Zhang J, Sun P, Yao C, Su C, Sui T, Huang H, Cao X, Ge Y (2010). EIF2alpha and caspase-12 activation are involved in oxygen-glucose-serum deprivation/restoration-induced apoptosis of spinal cord astrocytes. Neurosci Lett.

[6] Yu S, Liu M, Gu X, Ding F (2008). Neuroprotective effects of salidroside in the PC12 cell model exposed to hypoglycemia and serum limitation. Cell Mol Neurobiol.

[7] Huang SL, He XJ, Li ZF, Lin L, Cheng B (2014). Neuroprotective effects of ginsenoside Rg1 on oxygen-glucose deprivation reperfusion in PC12 cells. Pharmazie.

[8] Liu Z, Huang YY, Wang YX, Wang HG, Deng F, Heng B, Xie LH, Liu YQ (2015). Prevention of cell death by the zinc ion chelating agent TPEN in cultured PC12 cells exposed to Oxygen-Glucose Deprivation (OGD). J Trace Elem Med Biol.

[9] Vavilis T, Delivanoglou N, Aggelidou E, Stamoula E, Mellidis K, Kaidoglou A, Cheva A, Pourzitaki C, Chatzimeletiou K, Lazou A, Albani M, Kritis A (2016). Oxygen-glucose deprivation (OGD) modulates the unfolded protein response (UPR) and inflicts autophagy in a PC12 hypoxia cell line model. Cell Mol Neurobiol.

[10] Li C, Liu Y, Tang P, Liu P, Hou C, Zhang X, Chen L, Zhang L, Gu C (2016). Hydrogen sulfide prevents OGD/R-induced apoptosis by suppressing the phosphorylation of p38 and secretion of IL-6 in PC12 cells. Neuroreport.

[11] Mo ZT, Fang YQ, He YP, Zhang S (2012). beta-Asarone protects PC12 cells against OGD/R-induced injury via attenuating Beclin-1-dependent autophagy. Acta Pharmacol Sin.

[12] Zlobovskaya OA, Sergeeva TF, Shirmanova MV, Dudenkova VV, Sharonov GV, Zagaynova EV, Lukyanov KA (2016). Genetically encoded far-red fluorescent sensors for caspase-3 activity. BioTechniques.

[13] Wu XM, Chen HS, Jiang ZL, Zhu L, Jin SY (1999). Establishment of a hypoxia model of mouse cortical neurons and some observations. Acta Acad Med Nantong.

[14] Wu LY, Ding AS, Zhao T, Ma ZM, Wang FZ, Fan M (2005). Underlying mechanism of hypoxic preconditioning decreasing apoptosis induced by anoxia in cultured hippocampal neurons. Neurosignals.

[15] Nakagawa T, Zhu H, Morishima N, Li E, Xu J, Yankner BA, Yuan J (2000). Caspase-12 mediates endoplasmic-reticulum-specific apoptosis and cytotoxicity by amyloid-beta. Nature.

[16] Suzuki K, Imajoh S, Emori Y, Kawasaki H, Minami Y, Ohno S (1987). Calcium-activated neutral protease and its endogenous inhibitor. Activation at the cell membrane and biological function. FEBS Lett.

[17] Hitomi J, Katayama T, Taniguchi M, Honda A, Imaizumi K, Tohyama M (2004). Apoptosis induced by endoplasmic reticulum stress depends on activation of caspase-3 via caspase-12. Neurosci Lett.

[18] Thornberry NA, Lazebnik Y (1998). Caspases: enemies within. Science.

[19] Thornberry NA, Rano TA, Peterson EP, Rasper DM, Timkey T, Garcia-Calvo M, Houtzager VM, Nordstrom PA, Roy S, Vaillancourt JP, Chapman KT, Nicholson DW (1997). A combinatorial approach defines specificities of members of the caspase family and granzyme B. Functional relationships established for key mediators of apoptosis. J Biol Chem.

[20] Snigdha S, Smith ED, Prieto GA, Cotman CW (2012). Caspase-3 activation as a bifurcation point between plasticity and cell death. Neurosci Bull.

[21] Srinivasula SM, Fernandes-Alnemri T, Zangrilli J, Robertson N, Armstrong RC, Wang L, Trapani JA, Tomaselli KJ, Litwack G, Alnemri ES (1996). The Ced-3/interleukin 1beta converting enzyme-like homolog Mch6 and the lamin-cleaving enzyme Mch2alpha are substrates for the apoptotic mediator CPP32. J Biol Chem.

[22] Kuida K, Zheng TS, Na S, Kuan C, Yang D, Karasuyama H, Rakic P, Flavell RA (1996). Decreased apoptosis in the brain and premature lethality in CPP32-deficient mice. Nature.

[23] Fan W, Dai Y, Xu H, Zhu X, Cai P, Wang L, Sun C, Hu C, Zheng P, Zhao BQ (2014). Caspase-3 modulates regenerative response after stroke. Stem Cells.

